# The long shadow of socioeconomic deprivation over the modern management of acute myeloid leukemia: time to unravel the challenges

**DOI:** 10.1038/s41408-021-00533-0

**Published:** 2021-08-06

**Authors:** M. Joseph John, Philip Kuriakose, Mark Smith, Eve Roman, Sudhir Tauro

**Affiliations:** 1 Department of Clinical Haematology, Haemato-Oncology & Bone Marrow (Stem Cell) Transplantation, Christian Medical College, Ludhiana, Punjab India; 2 Division of Hematology and Oncology, Henry Ford Cancer Institute, Henry Ford Hospital, Detroit, MI USA; 3 Department of Haematology, Canterbury District Health Board, PO Box 151, Christchurch, New Zealand; 4 Department of Health Sciences, University of York, York, UK; 5 Department of Haematology and Division of Molecular & Clinical Medicine, Ninewells Hospital & School of Medicine, Dundee, UK

**Keywords:** Acute myeloid leukaemia, Therapeutics

## Abstract

Biological and non-biological variables unrelated to acute myeloid leukemia (AML) preclude standard therapy in many settings, with “real world” patients under-represented in clinical trials and prognostic models. Here, using a case-based format, we illustrate the impact that socioeconomic and anthropogeographical constraints can have on optimally managing AML in 4 different healthcare systems. The granular details provided, emphasize the need for the development and targeting of socioeconomic interventions that are commensurate with the changing landscape of AML therapeutics, in order to avoid worsening the disparity in outcomes between patients with biologically similar disease.

## Introduction

Until relatively recently, the drug therapy of non-promyelocytic acute myeloid leukemia (AML) had remained unchanged for over 40 years [1, 2]. An improved understanding of the molecular heterogeneity of AML [3, 4], recognition of disease with “actionable” targets [5] and the development of enhanced drug-delivery systems [6] are now yielding novel pharmacological approaches to treatment. As monotherapy, the effectiveness of these drugs is mostly restricted to modest increments in disease control [7–9], but when positioned appropriately with existing treatments, including stem cell transplantation, a clinically meaningful improvement in outcomes appears to be emerging [6, 10, 11]. The treatment paradigm for AML thus continues to evolve, informed by therapeutic intervention relevant to disease biology [12, 13] and in clinical trials aiming to standardize prognostication and, measurable residual disease (MRD) assessment, risk-adapt treatments, and improve survival [14].

Amidst this optimism, there remains a need to be cognizant of the many non-biological factors (NBF) unrelated to AML [15, 16] that could make the delivery of advances to many patients challenging. NBF include variations in levels of socioeconomic development, education, access to services, alcohol addiction, and mental health, which combined with the sustainability of funding within healthcare systems, can become the bottleneck that determines the equity of healthcare for AML patients. The detrimental impact of some of these variables is exemplified by the disproportionate contribution from low and middle-income countries to cancer mortality statistics, with the true magnitude of the problem almost certainly underestimated due to the inaccessibility of diagnostic and treatment pathways to most patients [17–19].

Discrepancies in AML outcomes related to NBF are well documented even within countries with high gross national incomes [20–27], with a recent Surveillance, Epidemiology, and End Results (SEER)-based study demonstrating a concerning widening of the survival gap between Black and White patients in the USA [28]. Patients with significant NBF and associated multi-morbidity [16], and those from certain ethnic groups remain under-represented in clinical trials [29], with these variables not featuring in any prognostic model for AML. For strategies in personalized medicine to be truly effective, the possibility of ethnicity-associated variation in outcomes, even with similar genetic drivers of disease and therapy [28] requires systematic investigation, and the term “supportive care” requires redefinition to include assistance with outcome-affecting NBF.

Here, we have used four real patient exemplars to review NBF that can interfere with the delivery of optimal AML treatment in different healthcare systems. The use of the case-based format that clinicians are familiar with, helps illustrate the extent of the challenge in a more transparent manner than is possible with quantitative analyses. Overcoming the difficulties in each narrative, all unrelated to the biology of the disease, requires significant longer-term financial and human investment, along with fundamental changes to the definition of well-being and expectation, not only from health policymakers and physicians, but also from patients and the public.

## Patient 1

A 34-year-old, previously healthy Nigerian Ph.D. student, resident in New Zealand on a Student Visa with his wife and 1-year-old child, presented with pancytopenia and blast cells in his blood film. Physical examination was unremarkable. Bone marrow biopsy showed a myeloid maturation arrest, with 82% monoblasts expressing CD45, CD64, CD33, CD4, CD56, and HLA-DR, confirming a diagnosis of AML with monocytoid differentiation. Marrow cytogenetic analysis showed a normal 46, XY karyotype. Mutation analysis of PCR-amplified bone marrow DNA was negative for *NPM1* and *FLT3* gene variants, including *FLT3* internal tandem duplications (ITD) and tyrosine kinase domain mutations.

## Treatment options

Based on the initial work-up, this patient had “intermediate-risk AML” [30–32]. With anthracycline and cytarabine-based induction chemotherapy, he had a 60–80% chance of achieving morphological remission, but with a high relapse-risk and 5-year survival of approximately 50% [30–32]. The risk of relapse can be reduced through the pre-emptive use of allogeneic stem cell transplantation (alloSCT) in first complete remission (CR1), since a donor-versus-no donor comparison confers a survival advantage with alloSCT in younger patients [33]. However, the fiscal implications of alloSCT and potential for toxicity, including death, mean that the allocation of alloSCT in CR1 should be risk-adapted and only offered to those with a higher predicted relapse-risk [34, 35]. Strategies for further risk-stratifying “intermediate-risk” AML patients include the use of targeted next-generation sequencing (NGS) in diagnostic specimens to detect gene variants associated with relapse, or the quantification of MRD at different stages of treatment using genetic markers or multi-parametric flow-cytometry [36–38]. In this context, it is important to acknowledge challenges to the standardization, quantification, and interpretation of genomic and flow-cytometry MRD that may limit the widespread adoption of MRD assessment as a prognostic tool [39]. Prospective randomized confirmation of the reduction in relapse-risk through post-remission intensification or alloSCT, based on mutational load and genomic profile at diagnosis, or MRD status, is required before MRD-adapted therapy becomes standard practice. In the absence of MRD measurements, validated scoring systems [40] and comorbidity [41] can be used to aid informed decision-making on alloSCT.

## Management and outcome

As a non-citizen, the patient did not automatically qualify for publicly funded AML treatment in New Zealand. He considered the low likelihood that he would receive appropriate treatment if he returned to his native Southern Nigeria, where he understood neither induction chemotherapy nor alloSCT was available. Therefore, he applied for leukemia treatment funding under his healthcare insurance policy in New Zealand and received approval for a maximum of four chemotherapy cycles, specifically excluding costs of alloSCT. Consequently, the use of molecular genomic risk-based or MRD-based allocation to alloSCT was not an option for this patient, whose treatment was determined solely by his insurance policy funding limitation that excluded alloSCT at any time.

He started intensive induction therapy with daunorubicin (60 mg/m^2^ on days 1, 3, and 5) and cytarabine (100 mg/m^2^) administered 12 hourly for 10 days (DA(60) 3+10) [14]. Bone marrow biopsy on day 29 of treatment following count recovery showed blasts of 8% indicating partial remission. Based on this level of cytoreduction, there would be no obvious benefit with alloSCT, particularly if CR is achieved after another cycle of induction therapy [40, 42]. He proceeded to his second cycle of treatment with 50 mg/m^2^ of daunorubicin on days 1, 3, and 5, and 8 days of cytarabine (100 mg/m^2^) administered 12 h apart (D(50)A 3+8) with a plan for consolidation with two cycles of high-dose cytarabine (3 g/m^2^), followed by an expectant observational strategy. Importantly, his visa status did not support public-funded remission re-induction followed by alloSCT in the event of relapse.

## Comments

The World Health Organisation (WHO) recognizes healthcare coverage as a global problem for the increasing number of international migrants and refugees in their draft global action plan, 2019-2023 [43]. In New Zealand, a refugee or “protected person” is covered by publicly funded health and disability services, but a full fee-paying student on a Student Visa is not eligible for state-funded healthcare and is required to hold healthcare insurance. The patient is thus responsible for healthcare costs that are not covered by insurance.

If insurance for healthcare costs is limited or absent, the personal responsibility to fund accumulating costs can be stressful for the patient and become a challenge for the hospital’s Eligibility Team. Life-saving interventions are provided regardless of a patient’s ability or willingness to pay, but cost recovery remains an expectation. Elective interventions to mitigate relapse-risk, for example, are not offered as publicly funded options. It is therefore important to indicate the costs of therapy to the patient early on during discussions regarding treatment. Despite language barriers that often compound the situation, clarity of communication is vital to emphasize the financial investment required to provide care that meets the country’s expected quality standard and is considered essential and life-saving. Permanent or temporary migrants, particularly fee-paying students, by the mere nature of their experiences are amongst those who would understand the nuances associated with the costs of treatment. If they originate from parts of the world where care is less than optimal, they could end up feeling victims of the system in the country of residence, as well as the place they consider “home”.

## Patient 2

A 30-year-old self-employed man, recently married, and with an income of less than US$ 285 per month presented with breathlessness, fever and leg pain to the hospital Emergency Department in Ludhiana, India. He had low hemoglobin (62 g/L), white cell count of 68.3 × 10^9^/L (predominantly blasts), and a platelet count of 259 ×10^9^/L. Coagulation screen, renal and liver function were normal. The bone marrow contained >80% blasts expressing CD34, HLA-DR, CD33, CD13, CD11b, and CD117 indicating a diagnosis of AML. Cytogenetic analysis was normal, and multiplex polymerase chain reaction did not detect the fusion gene re-arrangements *CBFβ-MYH11*, *RUNX1-RUNX1T1*, *PML-RARA*, *BCR-AB1*, or mutations in *NPM1*, *FLT3*, *c-KIT*, or *CEBPA*. The estimated costs for the diagnostic work-up including genetic testing was estimated at US$ 430.

## Treatment options

As with the previous case, this patient had intermediate-risk AML, but possibly with a greater relapse-risk on account of the higher white count [40]. He would be a candidate for standard induction therapy with an anthracycline and cytarabine backbone with post-remission therapeutic options assessed either through additional NGS or MRD studies. However, the Indian healthcare system is mainly reliant on patient-funded therapy and supportive care, making consideration of cost the starting-point of the treatment algorithm. For this patient, the cost of induction therapy was estimated to be between US$ 7150 and 11,450. For managing persistent fever and blood product support, the daily costs including hospital charges would be between US$ 214 and 285. The high incidence of invasive fungal infections, with the likelihood of requiring liposomal amphotericin, escalates the cumulative cost by at least 25% [44]. If remission is achieved, consolidation therapy with 3 cycles of cytarabine (3 g/m^2^) is estimated at US$ 20,200 in total, and alloSCT is expected to cost an additional US$ 14,300–21,450. The expected incidence of induction deaths is 25% [45]. The patient was offered induction treatment with daunorubicin (60 mg/m^2^/day for 3 days) and infusional cytarabine (100 mg/m^2^/day) for 7 days (DA(60) 3+7) [46] and subsequent consideration of alloSCT in remission, pending identification of an HLA-matched family donor and additional funding.

## Management and outcome

Following treatment with “DA(60) 3+7”, he required antibiotics for transient febrile neutropenia. On day +15, his white cell count increased to 9.8 × 10^9^/L with >90% of blasts. The kinetics of disease re-emergence with no reduction in the proportion of circulating blasts suggested primary refractory disease and the likely futility of a repeating induction treatment [42]. Treatment-intensification with fludarabine-based chemotherapy (FLAG-Ida) followed by consolidation with alloSCT was considered pending tissue-typing.

The re-calculated costs for FLAG-Ida alone, or in sequence with alloSCT in aplasia was estimated between US$ 14,300 and 35,714. Having to source this amount of money, for an estimated 20% chance of cure with the proposed treatment, was not an acceptable trade-off for the patient or his family. He expressed a desire to try an alternative, affordable treatment to prolong life and was offered treatment with subcutaneous azacitidine. However, he defaulted from follow-up less than 4 weeks into the diagnosis.

## Comments

In the Indian healthcare system, discussions around estimated treatment expenses and outcomes, based on biological parameters relevant to AML are critical before therapy, as physicians and patients attempt to balance the affordability of treatment with expected survival. This balance, a key determinant of treatment decisions becomes the proxy for cost-effectiveness, individual to each patient’s financial circumstances.

With a population approaching 1.4 billion, the Total Health Expenditure (THE) for India is estimated at US$ 70 billion (3.84% of GDP and US$ 54.40 per capita). The healthcare system is characterized by the co-existence of public and private health centers, poor public health infrastructure, high health care costs, and low insurance coverage [47]. The proportional contribution of patient-funded, out-of-pocket expenditure (OOPE) to THE is 60.6%, with smaller contributions from the government (22.8%), insurance (4.8%) and external donors including non-government organizations (11.8%) [47, 48]. Typical forms of distress financing to deal with the increased OOPE on health care include current income, savings, mortgaging and selling of assets, loans from moneylenders and financial institutions, and reduction in consumption expenditure [49, 50]. The adverse consequences of borrowing and selling assets to meet OOPE have significant short-term and longer-term consequences [51, 52]. Thus, the median age of AML patients treated in large centers in India is as low as 40 years, with less than a third of patients opting for induction chemotherapy, and standard-of-care treatments offered only to those with the potential to complete therapy [45].

## Patient 3

A 65-year-old African-American male living in the urban community in Detroit, USA, presented to an “outside” hospital with an infected right toe in January 2020 and a leucoerythroblastic blood film. He had retired 5 years previously, lived with his mother, and had an ECOG performance status of 2. Past medical history was significant for morbid obesity and obstructive sleep apnea requiring non-invasive ventilation, hypertension, non-insulin-dependent diabetes mellitus, and gout. Due to persisting infection and risk of overwhelming sepsis, he underwent amputation of the toe, following which he developed renal failure. Intravenous hydration caused circulatory overload and respiratory failure. Due to worsening leukocytosis, bone marrow examination was undertaken and AML was identified. He was transferred to a specialist unit with low hemoglobin (68 g/L) and platelet count (37 × 10^9^/L), with a white cell count of 79.3 × 10^9^/L and excess monoblasts and promonocytes. Repeat marrow examination confirmed monoblastic AML by morphology and immunophenotyping. Results of cytogenetic analysis received after the patient had started leukemia therapy identified inv [16] (p13.1q22), confirmed by interphase *FISH* in 85% of cells. NGS detected a low-level variant in *NRAS* and *KIT*.

## Treatment options

Based on age, patients with AML often end up being categorized as either “fit” or “unfit” to help determine the intensity of induction chemotherapy [53]. The presence of inv [16] is a “good risk” cytogenetic abnormality in AML, predicting a good response to intensive chemotherapy, particularly in patients younger than 60 years [30–32]. While remission rates in older patients are comparable, the relapse-risk is higher, with lower overall survival [54]. The addition of Mylotarg/gemtuzumab ozogamicin (GO), a humanized anti-CD33 monoclonal IgG4 antibody conjugate with calicheamicin to chemotherapy has been shown to associate with superior event-free and overall survival in AML [55–58]. Younger patients with “good risk” cytogenetic abnormalities are thought to derive particular benefit [55, 56], but the low incidence of this genetic subset in older patients makes the risk-benefit analysis of GO in patients ≥60 years of age with inv [16] AML less clear [57, 59]. Nevertheless, the use of GO with intensive chemotherapy is considered the standard for disease characterized by this chromosomal lesion [53], and the timely turnaround of genetic results is critical to inform optimal therapy with licensed targeted drugs or clinical trial participation.

Despite conflicting data, the presence of molecular genetic lesions, including concomitant mutations in *KIT* and *NRAS* may influence relapse following chemotherapy [60–63]. Whether intensifying therapy through alloSCT in CR1 in disease with a low burden of mutant alleles will improve outcomes, is unclear, but the measurement of fusion gene (*CBFB-MYH11*) transcripts as a marker of MRD, at pre-defined time-points during chemotherapy can be used to identify those at higher risk of relapse and guide therapeutic interventions including alloSCT [64]. Whether serial MRD monitoring to guide pre-emptive therapy will improve prognosis over offering treating at the point of hematological relapse, and outweigh the organizational and technical investment for MRD measurement and periods of potential inconvenience and anxiety to patients, is being investigated in the UK AML-17 trial for younger patients [14].

## Management and outcome

Based on frailty at the time of inter-hospital transfer and multi-morbidity pre-dating AML diagnosis, it was doubtful whether the patient would tolerate intensive chemotherapy. By optimizing fluid management, his organ function returned to baseline; based on leucocytosis mandating urgent treatment and normal cardiac imaging, he was offered intensive induction chemotherapy before the cytogenetic results were available. He consented to participate in phase II/III clinical trial investigating an experimental drug, administered with “DA(60) 3+7”. Randomized to the experimental arm, his tolerance of induction treatment was good. The day 14 bone marrow specimen identified resistant blasts (19%) but represented a >50% reduction in disease-burden over the diagnostic biopsy. Complications following re-induction therapy included culture negative neutropenic fever and respiratory distress, but invasive ventilation was not required. Coinciding with blood count recovery and attainment of CR, he became afebrile and oxygen-independent, although required transfer to an inpatient rehabilitation facility due to deterioration in mobility, functional and psychological status.

In view of the higher relapse-risk due to age and concurrent mutations in *KIT* and *NRAS* [63], the option of unrelated donor alloSCT as a post-remission strategy was subsequently discussed with the patient. He declined due to concerns regarding physical and psychological tolerance of treatment and suboptimal family support. Since the addition of GO was not permitted as per trial protocol, he was scheduled to continue on study for a total of three consolidation cycles with the investigational drug and high-dose cytarabine (2 g/m^2^), with observational follow-up. The alternative strategy of withdrawing him from the trial and treating him with GO-containing consolidation was not pursued. Since multi-morbidity and psychological frailty excluded alloSCT as a viable pre-emptive management strategy, MRD assessment through serial quantification of *CBF-MYH11* transcripts was not undertaken.

## Comments

Rapid changes in urbanization can negatively impact the economic and social structures of inner-city communities, adversely affecting health and quality of life [65]. Currently, over half of the world’s population lives in urban areas, and this is increasing markedly year-on-year, notably in less economically developed regions of the world [66]. Conversely, in more economically developed countries, the population decrease in previously affluent urban industrial areas has worsened the living conditions of those left behind [67, 68]. With a population of 1.85 million in 1950, Detroit was America’s fifth-largest city, but on account of human migration, was ranked closer to 20th by 2016, with a residual population of under a million. Similar to many midwestern and eastern cities in the USA, Detroit lost middle class and well-to-do families to its suburbs, taking away both people and jobs. The resulting restrictive business practices contributed to overcrowding and physical deterioration of neighborhoods within the city, similar to the predicament in other cities [66–69]. Soon, the multi-morbidity associated with deprivation that extends beyond measures of socioeconomic status, begins to define the neighborhood and interferes with the care of individuals, particularly those under the age of 70 years [70–72].

## Patient 4

A 56-year-old man presented to a hospital in Dundee, Scotland, UK with a brief history of progressive shortness of breath. Blood work demonstrated: hemoglobin 54 g/L, white cell count of 2.0 × 10^9^/L, neutrophil count of 0.4 × 10^9^/L, and platelet count of 52 × 10^9^/L, with normal coagulation screen. Bone marrow was infiltrated with blasts (72%) expressing CD33, CD13, CD117, and myeloperoxidase, indicating a diagnosis of AML. Cytogenetic analysis was normal and molecular analysis identified mutant N*PM-1* and wild-type *FLT3*. Renal and liver function were normal, while comorbidity included mild chronic obstructive airways disease and previous history of alcohol-dependence syndrome.

## Treatment options

Approximately 30% of patients with AML have a detectable mutation in nucleophosmin-1 (*NPM-1*) [73]. The frequency is higher in cytogenetically normal disease, and the prognosis with standard intensive chemotherapy is good in younger patients and those without a co-existing high *FLT3-*ITD burden [74, 75]. The fidelity of mutant *NPM-1* in most patients makes it a useful marker to quantify molecular MRD responses at pre-defined time-points following therapy to identify patients in CR1 who require treatment intensification for cure [76, 77]. More recently, lower-intensity protocols combining venetoclax with low-dose cytarabine or azacitidine [78, 79] have resulted in durable responses in *NPM-1*^mutant^ AML even in patients with molecular persistence or relapse following standard intensive chemotherapy [79]. On-going clinical trials aim to establish whether venetoclax-containing lower-intensity therapy should become the standard approach for all patients with *NPM-1*^mutant^ FLT3^wt^ AML.

## Management and outcome

This patient was offered intensive induction therapy with “DA(60) 3+10” [14]. Although he had no significant problems with his physical health, he was known to the psychiatric services since the age of 11 years, when he was evaluated for encopresis. In his 20 s, he was diagnosed with depressive neurosis and a schizoid personality disorder, revised to an “obsessional disorder”. He had been receiving help with anxiety management but defaulted from follow-up. He lived alone, was unemployed, and received social support from his daughter and a local priest. Over a 10-year period pre-dating the diagnosis of AML, he had contacted the emergency health services over 100 times for non-specific symptoms. He tended to self-discharge after hospitalization; eventually, his symptoms were attributed to somatization.

Despite the apparent understanding of the poor prognosis associated with untreated AML (confirmed by liaison psychiatry) the patient declined intensive chemotherapy in favor of supportive care and was discharged from the hospital. As an outpatient, he received treatment with subcutaneous low-dose cytarabine at a dose of 40 mg/day for 10 days. At the end of cycle 1, the blood count normalized to suggest remission. The patient expressed a desire to receive intensive chemotherapy, “to be able to remain alive”, thus demonstrating awareness of the non-curative effects of low-dose cytarabine. Following discussions involving the patient, liaison psychiatry, the patient’s daughter, priest, and family practitioner, a Hickman line was inserted to deliver intravenous cytarabine (1 g/m^2^ 12 hourly on alternate days, for a total of 6 doses). As the Hickman line was being accessed to administer the first dose of cytarabine, he decided against proceeding with any form of treatment. Disease relapse followed and the patient died within 6 months.

## Comments

Patients receiving a cancer diagnosis often experience a reactive deterioration in mental health [80]. However, as exemplified by this patient, poor mental health pre-dating the diagnosis of AML can add to the challenge of delivering optimal anti-leukemic therapy despite access to the publicly funded National Health Service Scotland. Socioeconomically, Dundee is a relatively deprived area of the country [81, 82], with around 40% of neighborhoods falling into the 20% most deprived areas in Scotland. Indeed, a significant proportion of the local population resides in areas with high death rates due to cancer, cardiovascular causes, drugs, and alcohol, and are three times more likely than those living in affluent areas to have below-average mental well-being. The cancer incidence and mortality rates in Scotland’s most deprived areas are, respectively, 32% and 74% higher than the least deprived areas, with a trend toward increasing mortality from the least deprived to most deprived areas in patients with leukemias [83]. Clearly, as well as physical health, poor mental health, and socioeconomic deprivation [16, 84, 85], both of which are associated with poor health service engagement and poor cancer outcomes, require targeting with more support than is currently provided.

## Discussion

The cases discussed here serve as a stark reminder of the challenges to be addressed proactively if AML patients worldwide are to benefit from improved diagnostic and disease-management strategies. Socioeconomic deprivation lies at the crux of many disparities, whether it be drug costs and the financing of supportive care, evident in the patient exemplars from India and New Zealand, or the physical or mental consequences of long-term social deprivation in the patient narratives from the USA and UK. Regrettably, there are few immediate solutions for optimizing the care of these patients, a fact that may seem incredulous to clinicians inexperienced in managing patients from deprived backgrounds: indeed, it would be easy to overlook subconsciously, the absence of interchangeability between the provision of disease-centered care and patient or people-centric management. The importance of implementing proposals aiming to reduce disparities in care therefore cannot be overemphasized.

Initiatives that could improve access to drugs include promoting value-based control of drug pricing, standardizing drug costs to national gross domestic product, and practice guidelines based on the best level of evidence [86]. For example, in the UK, health technology appraisal of new drugs is undertaken to determine “value-for-money” before the single-payer health system decides to fund the drug. Drug costs can also be reduced through the administration of lower drug doses, provided the efficacy of therapy is not compromised: in chronic myeloid leukemia (CML), many patients with excellent disease control can be safely maintained on a de-escalated dose of tyrosine kinase inhibitor [87]. The disease kinetics in AML requires a different approach, with an investigation of the pharmacogenomic determinants of drug metabolism and levels, or multi-drug schedules that permit dose-reductions through pharmacokinetic interactions: as an example, in combination with anti-fungal azole drugs, a considerably lower dose of venetoclax can be administered thereby reducing drug costs [88]. The affordability of leukemic drugs can also be improved through changes to the duration and scope of patent protection, or implementation of differential pricing structures [86]. It would however be naïve to underestimate the complexity of the challenges involved in getting agreement from different organizations involved in policy-making, and as “social advocates”, hemato-oncologists have an important role in shaping decisions.

Relevant to the costs of supportive care with blood products and anti-microbial therapy in newly diagnosed patients and during post-treatment aplasia, high-income countries have strategies that support patients through this phase of treatment. In many low and middle-income regions, there remains a lack of diagnostic capability and treatment centers with infrastructure capable of providing high-intensity supportive care [89]. Overcoming this deficit requires both national and international initiatives that create a network of high-quality centers per head of population that are accessible, accountable and offer standardized care at affordable or subsidized costs. The Ayushman Bharat National Health Protection Mission is India is one such ambitious project aiming to reduce economic disparities in healthcare including cancer treatments [90].

One possible way of adapting the governance and delivery of care to communities is through decentralizing health to local or regional governments [89], and maintaining incidence and outcomes statistics for the devolved regions so that the need for additional resource can be identified. The Cancer Quality Performance Indicators (QPI) in Scotland (https://www.isdscotland.org/Health-Topics/Quality-Indicators/Cancer-QPI/) exemplify a national governance framework that supports and measures the performance of regional cancer centers against a pre-defined ’standard of care’ target in cancers including AML, aiming to improve patient experience and survival whilst “reducing variance and ensuring safe, effective and person-centered cancer care”. Using survival as the surrogate for measuring “performance” however has the potential to be misleading due to the rarity and heterogeneous nature of AML, but data on treatment-type and early mortality, linked to patient demographics can help develop an understanding of disparities in therapy and unmet need. For example, publically available acute leukemia data on Scottish patients between 2014 and 2017 [91] have demonstrated areas for improvement: comprehensive diagnostic work-up was not undertaken in >25% of patients treated with curative intent, and access to clinical studies requires review, as <60% of patients received potentially curative treatment as trial participants. The QPI program, including the rationale for setting performance targets is not perfect and currently enables a comparison of regional rather than socioeconomic status-associated variation in healthcare, but the evolving model for egalitarian care that it provides should be lauded and adapted in other regions. Further steps towards providing equity of care include reviewing the two-tier framework that supports healthcare budgetary constraints, for example, in migrants faced with unexpected illness in New Zealand, and expanding the coverage of existing insurance schemes such as Medicaid, offering healthcare to the most deprived segments of society [92]. These initiatives should run in parallel with community educational programs that improve awareness of the importance of early diagnosis and treatment, common to all cancers.

When the affordability of a drug, or treatment costs are not a major barrier to therapy, the consequences of established physical or mental comorbidity, or behavioral issues that reflect protracted social deprivation become obstacles, as seen in the patients from the USA and UK. In patients unwilling to engage regularly with the healthcare system, interventions akin to those trialed with variable levels of success in public health initiatives tackling alcoholism [93], smoking [94], or hepatitis C therapy [95] require consideration: the use of text-message or specific app-based interventions or increased involvement of community-based allied healthcare professionals may optimize treatment compliance, an issue of particular importance with the advent of newer, oral drugs that can increase patient empowerment, and yet be paradoxically less desirable in some patients. The physical consequences of deprivation that interfere with the decision regarding intensive genotoxic therapy of AML are often irreversible. However, the identification of newer drugs with acceptable toxicity profiles may offer suitable alternatives to these patients. Prevention of physical comorbidities associated with socioeconomically deprived environments requires a longer-term approach that includes regeneration schemes [96] and research on environmental enrichment [97] as a means of reversing attitudes and health. Initiatives that improve access to services and social care, support healthier lifestyles, reducing worklessness, and improve social mobility are all longer-term strategies to improve general health and well-being within a community.

It is perhaps utopian to expect an elimination of disparities in AML care, but the experience with the distribution of anti-retroviral medication for human immunodeficiency virus infection [17], and the global collaboration resulting in comparable outcomes in acute promyelocytic leukemia in lower and high-income countries [98] are reasons for cautious optimism about the ability to reduce disparities, provided the desire for change is indeed genuine. Traditionally, risk-stratification that is used to inform therapeutic intervention in AML has been founded on the measurement of biological parameters. It is now time for us to consciously acknowledge socioeconomic and anthropogeographical confounders of outcomes, be courageous in expanding patient participation in clinical trials, and include measurement of NBF to improve the accuracy of prognostication and the assessment of disease impact on societies.

## References

[1] Wheatley K (2002). SAB-a promising new treatment to improve remission rates in AML in the elderly?. Br J Haematol.

[2] Burnett AK. Treatment of acute myeloid leukemia: are we making progress? Hematol Am Soc Hematol Educ Program. 2012;2012:1–6. 10.1182/asheducation-2012.1.1 23233553

[3] Papaemmanuil E, Gerstung M, Bullinger L, Gaidzik VI, Paschka P, Roberts ND (2016). Genomic classification and prognosis in acute myeloid leukemia. N Engl J Med.

[4] Grimwade D, Ivey A, Huntly BJ (2016). Molecular landscape of acute myeloid leukemia in younger adults and its clinical relevance. Blood.

[5] Stone RM, Mandrekar SJ, Sanford BL, Laumann K, Geyer S, Bloomfield CD (2017). Midostaurin plus chemotherapy for acute myeloid leukemia with a FLT3 mutation. N Engl J Med.

[6] Lancet JE, Uy GL, Cortes JE, Newell LF, Lin TL, Ritchie EK (2018). CPX-351 (cytarabine and daunorubicin) liposome for injection versus conventional cytarabine plus daunorubicin in older patients with newly diagnosed secondary acute myeloid leukemia. J Clin Oncol.

[7] Perl AE, Martinelli G, Cortes JE, Neubauer A, Berman E, Paolini S (2019). Gilteritinib or chemotherapy for relapsed or refractory FLT3-mutated AML. N Engl J Med.

[8] Stone RM, DeAngelo DJ, Klimek V, Galinsky I, Estey E, Nimer SD (2005). Patients with acute myeloid leukemia and an activating mutation in FLT3 respond to a small-molecule FLT3 tyrosine kinase inhibitor, PKC412. Blood..

[9] Konopleva M, Pollyea DA, Potluri J, Chyla B, Hogdal L, Busman T (2016). Efficacy and biological correlates of response in a phase II study of venetoclax monotherapy in patients with acute myelogenous leukemia. Cancer Discov.

[10] DiNardo CD, Jonas BA, Pullarkat V, Thirman MJ, Garcia JS, Wei AH (2020). Azacitidine and Venetoclax in previously untreated acute myeloid leukemia. N Engl J Med.

[11] Wei AH, Döhner H, Pocock C, Montesinos P, Afanasyev B, Dombret H (2020). Oral azacitidine maintenance therapy for acute myeloid leukemia in first remission. N Engl J Med.

[12] Richard-Carpentier G, DiNardo CD (2019). Single-agent and combination biologics in acute myeloid leukemia. Hematol Am Soc Hematol Educ Program.

[13] DiNardo CD, Wei AH (2020). How I treat acute myeloid leukemia in the era of new drugs. Blood..

[14] Burnett AK, Hills RK, Russell N (2020). Twenty five years of UK trials in acute myeloid leukaemia: what have we learned?. Br J Haematol.

[15] Adler NE, Newman K (2002). Socioeconomic disparities in health: pathways and policies. Health Aff.

[16] Mair FS, Jani BD (2020). Emerging trends and future research on the role of socioeconomic status in chronic illness and multimorbidity. Lancet Public Health.

[17] Knaul FM, Atun R, Farmer P, Frenk J (2013). Seizing the opportunity to close the cancer divide. Lancet..

[18] World Health Organization. WHO report on cancer: setting priorities, investing wisely and providing care for all. World Health Organization. 2020. https://apps.who.int/iris/handle/10665/330745.

[19] Fidler MM, Bray F (2018). Global cancer inequalities. Front Oncol.

[20] Munro AJ (2005). Keynote comment: deprivation and survival in patients with cancer: we know so much, but do so little. Lancet Oncol.

[21] Sekeres MA, Peterson B, Dodge RK, Mayer RJ, Moore JO, Lee EJ (2004). Differences in prognostic factors and outcomes in African Americans and whites with acute myeloid leukemia. Blood..

[22] Bhayat F, Das-Gupta E, Smith C, McKeever T, Hubbard R (2009). The incidence of and mortality from leukaemias in the UK: a general population-based study. BMC Cancer.

[23] Østgård LSG, Nørgaard M, Medeiros BC, Friis LS, Schoellkopf C, Severinsen MT (2017). Effects of education and income on treatment and outcome in patients with acute myeloid leukemia in a tax-supported health care system: A National Population-Based Cohort Study. J Clin Oncol.

[24] Jabo B, Morgan JW, Martinez ME, Ghamsary M, Wieduwilt MJ (2017). Sociodemographic disparities in chemotherapy and hematopoietic cell transplantation utilization among adult acute lymphoblastic and acute myeloid leukemia patients. PLoS ONE.

[25] Le Floch AC, Eisinger F, D’Incan E, Rey J, Charbonnier A, Caymaris L (2020). Socioeconomic deprivation is associated with decreased survival in patients with acute myeloid leukemia. Cancer Epidemiol.

[26] Berger E, Delpierre C, Despas F, Bertoli S, Bérard E, Bombarde O (2019). Are social inequalities in acute myeloid leukemia survival explained by differences in treatment utilization? Results from a French longitudinal observational study among older patients. BMC Cancer.

[27] Ip K, Bedair K, Tauro S (2020). An exemplar population-based study to predict up-take of non-intensive therapies in acute myeloid leukaemia. Leuk Res.

[28] Bhatnagar B, Kohlschmidt J, Mrózek K, Zhao Q, Fisher JL, Nicolet D (2021). Poor survival and differential impact of genetic features of black patients with acute myeloid leukemia. Cancer Discov.

[29] Gopishetty S, Kota V, Guddati AK (2020). Age and race distribution in patients in phase III oncology clinical trials. Am J Transl Res.

[30] Grimwade D, Hills RK, Moorman AV, Walker H, Chatters S, Goldstone AH (2010). Refinement of cytogenetic classification in acute myeloid leukemia: determination of prognostic significance of rare recurring chromosomal abnormalities among 5876 younger adult patients treated in the United Kingdom Medical Research Council trials. Blood..

[31] Döhner H, Estey E, Grimwade D, Amadori S, Appelbaum FR, Büchner T (2017). Diagnosis and management of AML in adults: 2017 ELN recommendations from an international expert panel. Blood..

[32] Byrd JC, Mrózek K, Dodge RK, Carroll AJ, Edwards CG, Arthur DC (2002). Pretreatment cytogenetic abnormalities are predictive of induction success, cumulative incidence of relapse, and overall survival in adult patients with de novo acute myeloid leukemia: results from Cancer and Leukemia Group B (CALGB 8461). Blood..

[33] Cornelissen JJ, van Putten WL, Verdonck LF, Theobald M, Jacky E, Daenen SM (2007). Results of a HOVON/SAKK donor versus no-donoranalysis of myeloablative HLA-identical sibling stem cell transplantation in first remission acute myeloid leukemia in young and middle-aged adults: benefits for whom?. Blood..

[34] Koreth J, Schlenk R, Kopecky KJ, Honda S, Sierra J, Djulbegovic BJ (2009). Allogeneic stem cell transplantation for acute myeloid leukemia in first complete remission: systematic review and meta-analysis of prospective clinical trials. JAMA..

[35] Craddock C, Raghavan M (2019). Which patients with acute myeloid leukemia in CR1 can be spared an allogeneic transplant?. Curr Opin Hematol.

[36] Schuurhuis GJ, Heuser M, Freeman S, Béné MC, Buccisano F, Cloos J (2018). Minimal/measurable residual disease in AML: a consensus document from the European LeukemiaNet MRD Working Party. Blood..

[37] Freeman SD, Hourigan CS (2019). MRD evaluation of AML in clinical practice: are we there yet?. Hematol Am Soc Hematol Educ Program.

[38] Freeman SD, Hills RK, Virgo P, Khan N, Couzens S, Dillon R (2018). Measurable residual disease at induction redefines partial response in acute myeloid leukemia and stratifies outcomes in patients at standard risk without NPM1 mutations. J Clin Oncol.

[39] Hourigan CS, Gale RP, Gormley NJ, Ossenkoppele GJ, Walter RB (2017). Measurable residual disease testing in acute myeloid leukaemia. Leukemia..

[40] Wheatley K, Burnett AK, Goldstone AH, Gray RG, Hann IM, Harrison CJ (1999). A simple, robust, validated and highly predictive index for the determination of risk-directed therapy in acute myeloid leukaemia derived from the MRC AML 10 trial. United Kingdom Medical Research Council’s Adult and Childhood Leukaemia Working Parties. Br J Haematol.

[41] Sorror ML, Storb RF, Sandmaier BM, Maziarz RT, Pulsipher MA, Maris MB (2014). Comorbidity-age index: a clinical measure of biologic age before allogeneic hematopoietic cell transplantation. J Clin Oncol.

[42] Ferguson P, Hills RK, Grech A, Betteridge S, Kjeldsen L, Dennis M (2016). An operational definition of primary refractory acute myeloid leukemia allowing early identification of patients who may benefit from allogeneic stem cell transplantation. Haematologica..

[43] World Health Organization. Promoting the health of refugees and migrants: draft global action plan, 2019–23. WHO; 2019.

[44] Korula A, Abraham A, Abubacker FN, Viswabandya A, Lakshmi KM, Abraham OC (2017). Invasive fungal infection following chemotherapy for acute myeloid leukaemia-Experience from a developing country. Mycoses..

[45] Philip C, George B, Ganapule A, Korula A, Jain P, Alex AA (2015). Acute myeloid leukaemia: challenges and real world data from India. Br J Haematol.

[46] Rai KR, Holland JF, Glidewell OJ, Weinberg V, Brunner K, Obrecht JP (1981). Treatment of acute myelocytic leukemia: a study by cancer and leukemia group B. Blood..

[47] Mishra S, Mohanty SK (2019). Out-of-pocket expenditure and distress financing on institutional delivery in India. Int J Equity Health.

[48] National Health Accounts Estimates for India (Internet). 2018. https://main.mohfw.gov.in/sites/default/files/NHA_Estimates_Report_2015-16_0.pdf. Accessed 26 Apr 2020.

[49] Sauerborn R, Adams A, Hien M (1996). Household strategies to cope with the economic costs of illness. Soc Sci Med.

[50] Onarheim KH, Sisay MM, Gizaw M, Moland KM, Norheim OF, Miljeteig I (2018). Selling my sheep to pay for medicines–household priorities and coping strategies in a setting without universal health coverage. BMC Health Serv Res.

[51] Dercon S (2002). Income risk, coping strategies, and safety nets. World Bank Res Obs.

[52] Flores G, Krishnakumar J, O’Donnell O, Van Doorslaer E (2008). Coping with health‐care costs: implications for the measurement of catastrophic expenditures and poverty. Health Econ.

[53] Tallman MS, Wang ES, Altman JK, Appelbaum FR, Bhatt VR, Bixby D (2019). Acute myeloid leukemia, version 3.2019, NCCN Clinical Practice Guidelines in Oncology. J Natl Compr Canc Netw.

[54] Grimwade D, Walker H, Harrison G, Oliver F, Chatters S, Harrison CJ (2001). The predictive value of hierarchical cytogenetic classification in older adults with acute myeloid leukemia (AML): analysis of 1065 patients entered into the United Kingdom Medical Research Council AML11 trial. Blood..

[55] Burnett AK, Hills RK, Milligan D, Kjeldsen L, Kell J, Russell NH (2011). Identification of patients with acute myeloblastic leukemia who benefit from the addition of gemtuzumab ozogamicin: results of the MRC AML15 trial. J Clin Oncol.

[56] Hills RK, Castaigne S, Appelbaum FR, Delaunay J, Petersdorf S, Othus M (2014). Addition of gemtuzumab ozogamicin to induction chemotherapy in adult patients with acute myeloid leukaemia: a meta-analysis of individual patient data from randomised controlled trials. Lancet Oncol.

[57] Burnett AK, Russell NH, Hills RK, Kell J, Freeman S, Kjeldsen L (2012). Addition of gemtuzumab ozogamicin to induction chemotherapy improves survival in older patients with acute myeloid leukemia. J Clin Oncol.

[58] Lambert J, Pautas C, Terré C, Raffoux E, Turlure P, Caillot D (2019). Gemtuzumab ozogamicin for de novo acute myeloid leukemia: final efficacy and safety updates from the open-label, phase III ALFA-0701 trial. Haematologica..

[59] Amadori S, Suciu S, Stasi R, Salih HR, Selleslag D, Muus P (2013). Sequential combination of gemtuzumab ozogamicin and standard chemotherapy in older patients with newly diagnosed acute myeloid leukemia: results of a randomized phase III trial by the EORTC and GIMEMA consortium (AML-17). J Clin Oncol.

[60] Paschka P, Marcucci G, Ruppert AS, Mrózek K, Chen H, Kittles RA (2006). Adverse prognostic significance of KIT mutations in adult acute myeloid leukemia with inv(16) and t(8;21): a Cancer and Leukemia Group B Study. J Clin Oncol.

[61] Park SH, Chi HS, Min SK, Park BG, Jang S, Park CJ (2011). Prognostic impact of c-KIT mutations in core binding factor acute myeloid leukemia. Leuk Res.

[62] Ishikawa Y, Kawashima N, Atsuta Y, Sugiura I, Sawa M, Dobashi N (2020). Prospective evaluation of prognostic impact of KIT mutations on acute myeloid leukemia with RUNX1-RUNX1T1 and CBFB-MYH11. Blood Adv.

[63] Itzykson R, Duployez N, Fasan A, Decool G, Marceau-Renaut A, Meggendorfer M (2018). Clonal interference of signaling mutations worsens prognosis in core-binding factor acute myeloid leukemia. Blood..

[64] Yin JA, O’Brien MA, Hills RK, Daly SB, Wheatley K, Burnett AK (2012). Minimal residual disease monitoring by quantitative RT-PCR in core binding factor AML allows risk stratification and predicts relapse: results of the United Kingdom MRC AML-15 trial. Blood..

[65] Kuddus MA, Tynan E, McBryde E (2020). Urbanization: a problem for the rich and the poor?. Public Health Rev.

[66] Stimson GV. The future of global health is urban health. Lancet. 2013;382:1475. 10.1016/S0140-6736(13)62241-2 24182531

[67] European Commission. Increasing or declining urban populations - the future of cities, 2019. https://ec.europa.eu/jrc/en/facts4eufuture/future-of-cities/urban-populations; 2019.

[68] Kasarda JD, Appold SJ, Sweeney SH, Seiff E. Central-City and Suburban Migration Patterns: Is a Turnaround on the Horizon? Housing Policy Debate 8, no. 2 (January 1997): 307–58.

[69] Kruse KM. White flight: Atlanta and the making of modern conservatism. Princeton, N.J.: Princeton University Press; 2007.

[70] Thabit W. How east New York became a ghetto. New York: New York University Press; 2003. p. 42.

[71] Takahashi PY, Ryu E, Hathcock MA, Olson JE, Bielinski SJ, Cerhan JR (2016). A novel housing-based socioeconomic measure predicts hospitalisation and multiple chronic conditions in a community population. J Epidemiol Community Health.

[72] Chamberlain AM, Finney Rutten LJ, Wilson PM, Fan C, Boyd CM, Jacobson DJ (2020). Neighborhood socioeconomic disadvantage is associated with multimorbidity in a geographically-defined community. BMC Public Health.

[73] Arber DA, et al. Acute myeloid leukaemia with recurrent genetic abnormalities. In: Swerdlow SH, Campo E, Harris NL, Jaffe ES, Pileri SA, Stein H, et al., editors. WHO Classification of tumours of haematopoietic and lymphoid tissues. Lyon: IARC; 2017. p. 130–49.

[74] Falini B, Mecucci C, Tiacci E, Alcalay M, Rosati R, Pasqualucci L (2005). Cytoplasmic nucleophosmin in acute myelogenous leukemia with a normal karyotype. N Engl J Med.

[75] Schlenk RF, Döhner K, Krauter J, Fröhling S, Corbacioglu A, Bullinger L (2008). Mutations and treatment outcome in cytogenetically normal acute myeloid leukemia. N Engl J Med.

[76] Ivey A, Hills RK, Simpson MA, Jovanovic JV, Gilkes A, Grech A (2016). Assessment of Minimal Residual Disease in Standard-Risk AML. N Engl J Med.

[77] Dillon R, Potter N, Freeman S, Russell N (2021). How we use molecular minimal residual disease (MRD) testing in acute myeloid leukaemia (AML). Br J Haematol.

[78] Lachowiez CA, Loghavi S, Kadia TM, Daver N, Borthakur G, Pemmaraju N (2020). Outcomes of older patients with NPM1-mutated AML: current treatments and the promise of venetoclax-based regimens. Blood Adv.

[79] Tiong IS, Dillon R, Ivey A, Teh TC, Nguyen P, Cummings N (2021). Venetoclax induces rapid elimination of NPM1 mutant measurable residual disease in combination with low-intensity chemotherapy in acute myeloid leukaemia. Br J Haematol.

[80] Cordova MJ, Riba MB, Spiegel D (2017). Post-traumatic stress disorder and cancer. Lancet Psychiatry.

[81] Dundee City—Poverty Profile. https://www.dundeecity.gov.uk/service-area/chief-executive/chief-executives-services/dundee-partnership/publications/poverty-profile-2019, 2019.

[82] Long-term monitoring of health inequalities: January 2020 report. An official statistics publication for Scotland. https://www.gov.scot/publications/long-term-monitoring-health-inequalities-january-2020-report/; 2020.

[83] Cancer Mortality in Scotland Annual update to 2018. A national statistics publication for Scotland. https://www.isdscotland.org/Health-Topics/Cancer/Publications/2019-10-29/2019-10-29-Cancer-Mortality-Report.pdf; 2019.

[84] Kivimäki M, Batty GD, Pentti J, Shipley MJ, Sipilä PN, Nyberg ST (2020). Association between socioeconomic status and the development of mental and physical health conditions in adulthood: a multi-cohort study. Lancet Public Health.

[85] Saxena S, Thornicroft G, Knapp M, Whiteford H (2007). Resources for mental health: scarcity, inequity, and inefficiency. Lancet..

[86] Leighl NB, Nirmalakumar S, Ezeife DA, Gyawali B (2021). An arm and a leg: the rising cost of cancer drugs and impact on access. Am Soc Clin Oncol Educ Book.

[87] Clark RE, Polydoros F, Apperley JF, Milojkovic D, Pocock C, Smith G (2017). De-escalation of tyrosine kinase inhibitor dose in patients with chronic myeloid leukaemia with stable major molecular response (DESTINY): an interim analysis of a non-randomised, phase 2 trial. Lancet Haematol.

[88] Agarwal SK, DiNardo CD, Potluri J, Dunbar M, Kantarjian HM, Humerickhouse RA (2017). Management of venetoclax-posaconazole interaction in acute myeloid leukemia patients: evaluation of dose adjustments. Clin Ther.

[89] Pramesh CS, Badwe RA, Borthakur BB, Chandra M, Raj EH, Kannan T (2014). Delivery of affordable and equitable cancer care in India. Lancet Oncol.

[90] Caduff C, Booth CM, Pramesh CS, Sullivan R (2019). India’s new health scheme: what does it mean for cancer care?. Lancet Oncol.

[91] Acute Leukaemia Quality Performance Indicators Patients diagnosed between July 2014 and June 2017. https://www.isdscotland.org/Health-Topics/Quality-Indicators/Publications/2018-06-19/2018-06-19-Acute-Leukaemia-QPI-Report.pdf. An Official Statistics publication for Scotland; 2018.

[92] Rosenbaum S (2021). Health equity and medicaid transformation - operationalizing President Biden’s agenda. N Engl J Med.

[93] Crombie IK, Irvine L, Williams B, Sniehotta FF, Petrie D, Jones C (2018). Texting to Reduce Alcohol Misuse (TRAM): main findings from a randomized controlled trial of a text message intervention to reduce binge drinking among disadvantaged men. Addiction.

[94] Whittaker R, McRobbie H, Bullen C, Rodgers A, Gu Y, Dobson R (2019). Mobile phone text messaging and app-based interventions for smoking cessation. Cochrane Database Syst Rev.

[95] Radley A, de Bruin M, Inglis SK, Donnan PT, Hapca A, Barclay ST (2020). Clinical effectiveness of pharmacist-led versus conventionally delivered antiviral treatment for hepatitis C virus in patients receiving opioid substitution therapy: a pragmatic, cluster-randomised trial. Lancet Gastroenterol Hepatol.

[96] Greene G, Fone D, Farewell D, Rodgers S, Paranjothy S, Carter B (2020). Improving mental health through neighbourhood regeneration: the role of cohesion, belonging, quality and disorder. Eur J Public Health.

[97] Kühn S, Düzel S, Eibich P, Krekel C, Wüstemann H, Kolbe J (2017). In search of features that constitute an “enriched environment” in humans: Associations between geographical properties and brain structure. Sci Rep.

[98] Rego EM, Kim HT, Ruiz-Argüelles GJ, Undurraga MS, Uriarte Mdel R, Jacomo RH (2013). Improving acute promyelocytic leukemia (APL) outcome in developing countries through networking, results of the International Consortium on APL. Blood..

